# Treating Catatonia: a Blind Spot of Psychiatry?

**DOI:** 10.1192/j.eurpsy.2022.143

**Published:** 2022-09-01

**Authors:** P. Mohr

**Affiliations:** National Institute of Mental Health, Czech Republic, Nimh, Klecany, Czech Republic

**Keywords:** ICD-11, Treatment, Catatonia

## Abstract

Catatonia is a syndrome of primarily psychomotor disturbances associated with typical abnormalities of muscle tone. It is characterized by the co-occurrence of several symptoms of decreased, increased, or abnormal psychomotor activity. Catatonia is a neuropsychiatric syndrome, not an independent nosological entity. Historically associated mainly with schizophrenia (e.g., catatonic subtype), ICD-11, similarly to DSM-5, now recognizes catatonia under a separate classification category, apart from psychotic disorders. In addition to schizophrenia and other primary psychotic disorders, it can occur in the context of other mental disorders, such as mood disorders, or neurodevelopmental disorders, especially autism spectrum disorder. Catatonia can also develop during or immediately after intoxication or withdrawal from psychoactive substances, including phencyclidine, cannabis, hallucinogens such as mescaline or LSD, cocaine and MDMA or related drugs, or during the use of some psychoactive and non-psychoactive medications (e.g. antipsychotic medications, benzodiazepines, steroids, disulfiram, ciprofloxacin). Moreover, catatonia can occur as a direct pathophysiological consequence of various nonpsychiatric medical conditions, e.g., diabetic ketoacidosis, hypercalcemia, hepatic encephalopathy, homocystinuria, neoplasms head trauma, cerebrovascular disease, or encephalitis. Due to the fact that catatonia was mostly associated witch schizophrenia, many cases were not diagnosed and thus did not receive indicated treatment. There are no specific “anti-catatonic” drugs, first-line treatment are benzodiazepines and ECT, in addition to the symptomatic and supportive therapy. The recognition of catatonia as an independent category in ICD-11 can improve medical care for catatonic patients in clinical practice.

**Disclosure:**

No significant relationships.

